# Bidirectional Gated Recurrent Unit Neural Network for Fault Diagnosis and Rapid Maintenance in Medium-Voltage Direct Current Systems

**DOI:** 10.3390/s25030693

**Published:** 2025-01-24

**Authors:** Bohyung Lee, Yeseul Kim, Hyunyong Lee, Changmook Kang

**Affiliations:** 1 Department of Electrical Engineering, Hanyang University, Seoul 04763, Republic of Korea; bogud298@hanyang.ac.kr; 2 Department of Electrical Engineering, Incheon National University, Incheon 22012, Republic of Korea; oedam@inu.ac.kr; 3 Energy System Research Section, Honam Research Center, Electronics and Telecommunications Research Institute (ETRI), Daejeon 61012, Republic of Korea; hyunyonglee@etri.re.kr

**Keywords:** bidirectional gated recurrent unit (Bi-GRU), MVDC system, DC fault, fault diagnosis, deep learning

## Abstract

With the growing penetration of renewable energy sources, ensuring the stability and reliability of Medium-Voltage Direct Current (MVDC) systems has become more critical than ever. A single fault in MVDC systems can cause significant disturbances, necessitating rapid and precise diagnostics to prevent equipment damage and maintain continuous power supply. In this work, we present a Bidirectional Gated Recurrent Unit (Bi-GRU) model that both classifies and locates MVDC faults. By capturing the temporal behavior of voltage signals, the Bi-GRU framework surpasses traditional algorithms such as Convolutional Neural Networks (CNNs) and Bidirectional Long Short-Term Memory (Bi-LSTM) networks. Furthermore, the proposed approach addresses multiple fault scenarios including PTP (Pole-to-Pole), PPTG (Positive Pole-to-Ground), and NPTG (Negative Pole-to-Ground) while preserving real-time diagnostic capabilities. In extensive tests, our model achieves an overall accuracy of 95.54% and an average fault detection time below 1.3 ms, meeting real-world operational requirements. To assess robustness, sensor noise was artificially introduced to emulate realistic conditions. Despite these challenging inputs, our method consistently maintained high diagnostic accuracy, confirming its practicality and reliability. Consequently, the proposed scheme demonstrates a significant contribution toward improving the safety and dependability of MVDC systems, even under noisy conditions.

## 1. Introduction

Modern society faces critical global challenges such as environmental issues and energy depletion. These issues have significantly propelled the development and expansion of renewable energy solutions [1,2,3]. In particular, growing policy demands aimed at reducing carbon emissions and improving energy efficiency have accelerated the deployment of solar, wind and hydro resources [4,5]. Nonetheless, integrating such geographically dispersed and intermittently generated energy poses considerable technical hurdles. To overcome these obstacles, advanced and robust power transmission strategies are imperative [6].

In this context, Medium-Voltage Direct Current (MVDC) technology has emerged as a promising solution. MVDC systems operate at voltage levels ranging from a few kV to tens of kV (approximately 1 kV to 100 kV) and offer numerous advantages over traditional Alternating Current (AC) systems [7,8,9]. By increasing voltage levels, MVDC systems significantly minimize power losses during transmission, enabling greater efficiency and cost-effectiveness throughout the energy supply chain. Furthermore, MVDC systems exhibit high power density, making them ideal for space-constrained environments such as offshore platforms, ships, and industrial sites. Additionally, they are highly compatible with DC-based energy sources such as solar power and Energy Storage Systems (ESS). This capability enhances power quality and ensures the seamless integration of diverse renewable energy resources. As a result, MVDC systems effectively address the growing demand for reliable, high-quality electricity, particularly in medium-distance transmissions between coastal and urban areas or within industrial distribution networks [10,11].

**Figure 1:**
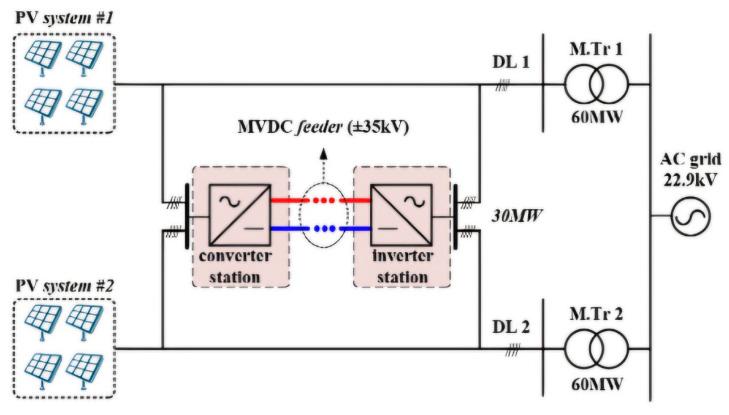
Configuration of hybrid MVDC distribution system.

As a practical example, South Korea’s ±35 kV MVDC station is undergoing demonstration research as part of a national project to verify the effectiveness of MVDC technology. This system, located in Naju, is designed with a two-terminal (Point-to-Point) configuration, as illustrated in Figure 1 [12]. This project focuses on integrating renewable energy sources, such as wind and solar power, into the national grid. Furthermore, MVDC systems can efficiently transmit power over medium distances while minimizing transmission losses. Furthermore, MVDC technology enhances grid reliability and supports the transition toward sustainable energy solutions. Such practical applications highlight the importance of MVDC systems in modern energy infrastructure and their role in shaping the future energy landscape [13].

Nonetheless, MVDC fault management introduces its own set of challenges. Unlike AC systems, the nature of faults in Direct Current (DC) systems involves distinct characteristics, necessitating specialized fault diagnosis and protection mechanisms [14,15]. The primary challenges stem from the types and characteristics of faults that occur in MVDC systems, such as PTP (Pole-to-Pole), PPTG (Positive Pole-to-Ground), and NPTG (Negative Pole-to-Ground). With PTP in DC systems, the fault begins with a rapid capacitor discharge, followed by the diode freewheel stage where current circulates, and finally transitions to grid-side current feeding, prolonging the fault’s duration. PPTG and NPTG, meanwhile, can result in severe equipment damage if left undetected, emphasizing the critical need for effective fault diagnosis mechanisms [16].

Moreover, the rapid rise in current characteristic of DC systems exacerbates the severity of faults, requiring immediate and accurate detection to prevent significant equipment damage and ensure grid stability. Compounding these challenges are the long transmission distances and diverse fault locations characteristic of MVDC systems, which produce varying voltage and current signal patterns. This variability complicates the design and implementation of effective fault diagnosis methods [17,18,19,20].

Fault diagnosis methodologies have significantly evolved over the decades, transitioning from traditional mathematical model-based approaches to modern data-driven techniques [21]. In the 1970s and 1980s, initial methods relied on analytical models that exploited differences between normal and fault conditions [22,23]. While effective for simpler systems, these approaches struggled to handle the nonlinear characteristics inherent in DC systems. By the 1990s, data-driven methods incorporating neural networks emerged, providing more adaptive diagnostic capabilities [24]. However, these methods required large datasets and faced challenges in simultaneously addressing fault types and locations. In the 2000s, signal processing techniques, such as wavelet transforms, enabled precise fault analysis in the time–frequency domain but lacked real-time diagnostic capabilities. This approach has limitations as it requires the manual determination of fault thresholds even after extracting fault features from signals, which hinders achieving sufficient diagnostic accuracy [25]. Deep learning models, including Convolutional Neural Networks (CNN) and Bidirectional Long Short-Term Memory (Bi-LSTM) networks, have demonstrated significant advancements in analyzing complex fault signals. These advancements in AI algorithms have been applied to system fault diagnosis, offering significant advantages over traditional DC fault detection methods. AI algorithms eliminate the need for manual threshold setting, leveraging large-scale data to prevent design errors and significantly improve diagnostic accuracy [26,27,28].

This study proposes a Bi-GRU-based model for fault diagnosis in MVDC systems. By leveraging the temporal modeling capabilities of Bi-GRU, this model enables the simultaneous diagnosis of fault types and locations, addressing these aspects as a multi-classification problem. The proposed method demonstrates superior accuracy, achieving a fault diagnosis accuracy of 95.54%, with robust performance against noise. Furthermore, the adoption of a sliding window mechanism ensures real-time fault detection, allowing the system to respond within milliseconds [29].

The main contributions of this research are the following:

1. The development of a Bi-GRU-based time-series analysis model to effectively capture the temporal characteristics of fault signals.
2. The introduction of a multi-diagnosis structure that simultaneously diagnoses fault types and locations, overcoming limitations of traditional methods.
3. The validation of noise robustness, ensuring the model’s practical applicability in real-world environments.
4. Comparative analysis with CNN and Bi-LSTM models, showcasing superior performance in terms of accuracy (95.54%), diagnosis time, and reliability.

This research aimed to resolve the unique challenges of MVDC systems, enhance grid reliability, and contribute to the broader adoption of renewable energy sources. By addressing these challenges, the proposed approach supports the development of sustainable and efficient power systems [30].

## 2. Simulation Model and Fault Definition

**Figure 2:**
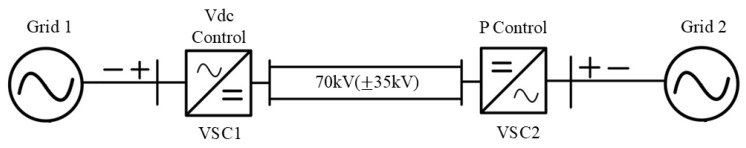
Topology diagram of the MVDC system.

A topology diagram of the MVDC system is illustrated in Figure 2. A two-terminal MVDC electromagnetic transient simulation model was developed using MATLAB/Simulink 2024b to replicate realistic operating conditions and fault scenarios. The model encompasses all key components of the MVDC system, ensuring the accurate simulation of fault characteristics and response.

In MVDC systems, the power converter control strategies can be categorized into Grid-Feeding Control and Grid-Forming Control [31,32]. Grid-Feeding Control operates under the assumption that the external AC grid, or an upper-level system, determines the voltage and frequency. The converter synchronizes with this reference using a Phase-Locked Loop (PLL), allowing it to regulate active and reactive power accordingly. This approach is relatively simple to implement and has demonstrated stability in commercial applications, making it a widely utilized choice in environments with a reliable external grid.

In contrast, Grid-Forming Control enables the inverter to independently generate voltage and frequency, thereby providing the reference for the power network itself. This strategy is especially valuable in scenarios with a high penetration of renewable energy sources, as it can flexibly supply reactive power and enhance system stability. However, the implementation of Grid-Forming Control poses challenges due to the need for complex control algorithms and precise parameter tuning.

In this study, the Grid-Feeding Control strategy was selected based on the assumption that the MVDC network receives its reference voltage and frequency from an upper-level AC grid. To achieve this, a PLL-based synchronization mechanism was combined with a DC voltage control loop and an inner current control loop. This integration simplifies the implementation and operation of the system while providing practical advantages for fault diagnosis and protection frameworks.

A 50 km DC transmission line was selected in this study to evaluate the economic and technical feasibility of MVDC systems in long-distance scenarios. Previous studies [33] have shown that MVDC systems become more cost-effective than AC systems when the transmission distance exceeds thresholds such as 17.5 km for large-scale applications and 29 km for smaller installations. By selecting 50 km, this study provides insights into MVDC system performance in extended applications.

Furthermore, the parameters listed in Table 1 are crucial for replicating realistic operational conditions and fault scenarios. The transmission line length can be adjusted to suit various application requirements.

### 2.1. Key Components and Configuration

The MVDC system was engineered to ensure the efficient transmission of power and robust fault diagnosis. The rectifier station converts AC to DC using a three-level voltage source converter (VSC), maintaining stable DC voltage ($V_{\mathrm{dc}}^{\mathrm{ref}}$) and integrating DC filters for noise suppression. The inverter station at the opposite terminal converts DC back to AC while managing active power flow ($P_{\mathrm{ac}}^{\mathrm{ref}}$) to ensure system stability under diverse operating conditions.

The 50 km DC transmission line is divided into 3–4 km sections using PI models. This segmentation facilitates localized fault detection and simplifies maintenance by pinpointing the exact fault location, significantly reducing downtime and repair efforts.

### 2.2. Fault Definition

In MVDC systems, faults are defined based on their impact on transmission performance and maintenance requirements. Key fault types include the following:

- Pole-to-Pole (PTP): A direct short circuit between the positive and negative poles, leading to rapid current surges and voltage collapse.
- Positive Pole-to-Ground (PPTG): A fault connecting the positive pole to the ground, resulting in an imbalance and potential overvoltage in the negative pole.
- Negative Pole-to-Ground (NPTG): A fault connecting the negative pole to the ground, causing similar challenges as PPTG.

While faults can occur in various components of MVDC systems, such as converters, inverters, and battery systems, this study focuses specifically on DC line faults. The DC line serves as the main power transmission path, and faults in this area are critical due to their potential to disrupt the overall system’s operation. By focusing on DC line faults, this approach enables rapid fault detection and maintenance, thereby improving system reliability and minimizing downtime. Additionally, this focus simplifies the fault diagnosis process and aids in developing effective protection strategies tailored to MVDC systems [34].

**Figure 3:**
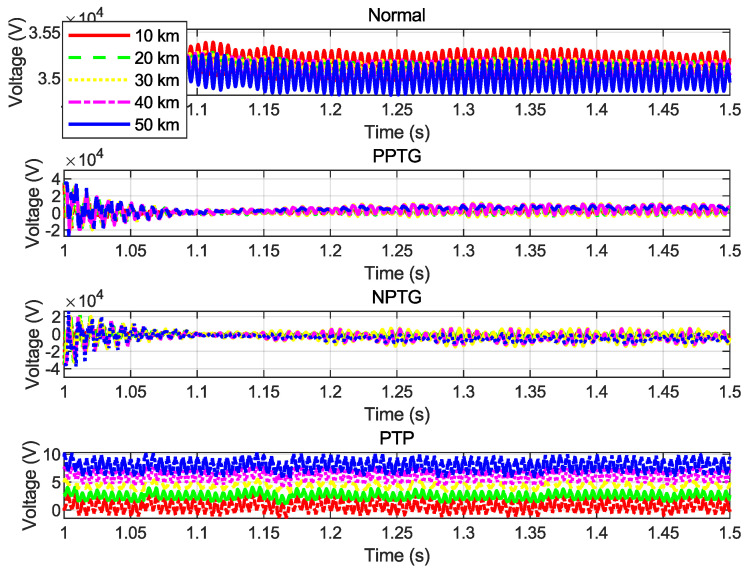
Voltage signal patterns at different distances: Normal, PPTG, NPTG, and PTP.

Figure 3 illustrates the voltage waveforms recorded during various fault scenarios. These waveforms enable quick and accurate fault diagnosis and localization, facilitating efficient preventive maintenance and minimizing the impact on overall system performance.

However, the voltage waveforms associated with different fault scenarios often exhibit overlapping patterns, making it challenging to distinguish between fault types using traditional analysis methods. To address this, BI-GRU was employed to efficiently analyze and classify fault signals. BI-GRU leverages its capability to capture both forward and backward dependencies in time-series data, enabling more accurate fault type identification and localization. This study emphasizes the significance of addressing DC line faults to ensure the stable and reliable operation of MVDC systems.

### 2.3. Incorporation of Sensor Noise

To simulate real-world conditions, band-limited white noise was added to voltage ($V_{P},V_{N}$) signals. The noise parameters included a power value of 0.001 and a sampling time of 0.01, replicating measurement inaccuracies inherent in practical systems. The inclusion of noise highlights the robustness of the proposed diagnostic model.

### 2.4. Fault Detection

The fault detection model employs time-domain analysis, leveraging the propagation characteristics of fault signals along the segmented DC line. Each location’s response to faults is analyzed to identify both the type and location of the fault. For validation, faults were generated at intervals of 3 km, 6 km, 10 km, and beyond, with testing performed on unseen data at specific points. This model ensures high accuracy and reliability across diverse fault scenarios.

**Figure 4:**
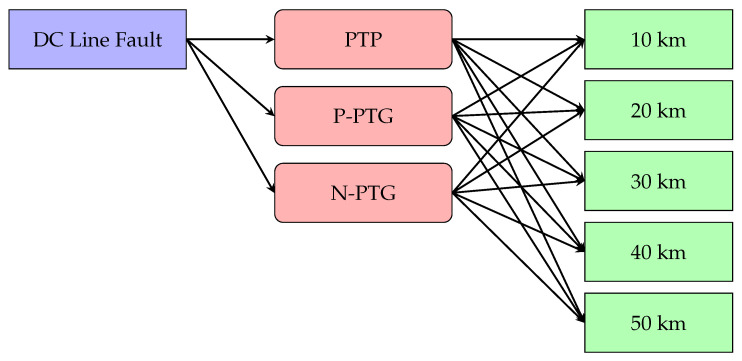
Diagram illustrating DC line fault types and their associated locations.

Figure 4 illustrates the relationship between DC line fault types and their corresponding locations. The diagram includes three fault types, PTP, PPTG, and NPTG, along with five DC line sections divided at intervals of 10 km, 20 km, 30 km, 40 km, and 50 km. When a fault occurs on the DC line, the model first identifies the fault type (PTP, PPTG, or NPTG) and then determines the fault’s location within a specific section (e.g., 10 km, 20 km, etc.). These fault types can occur in all sections, and the voltage and current signal characteristics vary depending on the fault type.

This study proposes a method for categorizing fault locations in MVDC systems based on predefined sections. This approach provides approximate fault locations, enhancing the efficiency of maintenance and repair operations while significantly reducing system recovery time.

The section-based approach provides information on both the fault type and the approximate fault section, enabling more targeted and efficient resource allocation. By focusing on specific sections, this method minimizes unnecessary equipment inspections and optimizes the deployment of maintenance personnel and tools.

A previous study [35] emphasized the advantages of segmenting DC lines into predefined sections to simplify fault handling and maintenance. Inspired by this concept, our study adopted a 10 km segmentation to demonstrate the practicality of this approach. This segmentation can be further refined or adjusted to meet specific application requirements, offering flexibility for various scenarios.

To support this analysis, a Bi-GRU-based model was employed. The Bi-GRU model leverages bidirectional information flow to process time-series data and efficiently learn the relationships between fault signal patterns and their locations. The model outputs a total of 16 classifications, corresponding to the combinations of three fault types (PTP, PPTG, NPTG) and five DC line segments (10 km, 20 km, 30 km, 40 km, 50 km), along with a “no fault” condition. Further details about the architecture and functionalities of the Bi-GRU model are described in Section 3.

## 3. Bi-GRU-Based Fault Detection for MVDC

The proposed fault detection model is based on the Bi-GRU network, which excels at analyzing temporal dependencies in time-series data [36]. Bi-GRU was specifically chosen due to its ability to address key limitations of traditional RNNs (Recurrent Neural Networks). In standard RNNs, issues such as vanishing and exploding gradients often arise during backpropagation, particularly when processing long sequences. These problems significantly hinder the learning of long-term dependencies in data. GRU, and by extension Bi-GRU, resolves these challenges by introducing gating mechanisms that effectively control the flow of information, allowing the model to retain relevant features over extended time steps.

Using the bidirectional processing capabilities of Bi-GRU, the model effectively captures both past and future contextual information in fault signals, enabling precise fault type classification and location detection. This capability is particularly crucial for MVDC systems, where transient and time-dependent fault characteristics significantly influence diagnostic accuracy.

One of the primary strengths of the Bi-GRU model lies in its efficient parameter usage compared to Bi-LSTM, without compromising performance. This efficiency makes it suitable for real-time fault diagnosis applications. Furthermore, the architecture’s robustness to noise ensures consistent performance in noisy and dynamic environments, addressing the challenges encountered in real-world MVDC systems. The model’s scalability allows seamless adaptation to longer input sequences or additional fault scenarios, highlighting its practicality and versatility.

The Bi-GRU network constitutes the core of the model, responsible for feature extraction and temporal modeling. Unlike traditional GRU, the Bi-GRU architecture processes input sequences in both forward and backward directions, allowing it to capture dependencies from both past and future time steps. This bidirectional flow of information is particularly advantageous for fault diagnosis, where signals preceding and following a fault event provide critical diagnostic information [36,37].

**Figure 5:**
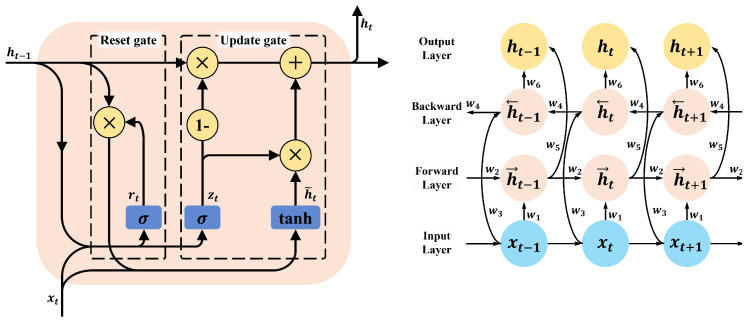
The structures of GRU and Bi-GRU.

Figure 5 illustrates the internal structure of a GRU and the architectural design of a Bi-GRU. The GRU incorporates gating mechanisms such as reset and update gates to control the flow of information, addressing the vanishing and exploding gradient issues encountered in traditional RNNs. The Bi-GRU extends this design by including both forward and backward layers, enabling the model to utilize bidirectional context effectively.

### 3.1. Forward and Backward GRU

At each time step *t*, the forward GRU computes a hidden state ($\vec{h_{t}}$) based on the current input ($x_{t}$) and the previous hidden state ($\vec{h_{t-1}}$):

$$\vec{h_{t}}=\mathrm{GRU}\left(x_{t},\vec{h_{t-1}}\right)$$

Simultaneously, the backward GRU computes a hidden state ($\overset{\leftarrow }{h_{t}}$) by processing the sequence in reverse order:

$$\overset{\leftarrow }{h_{t}}=\mathrm{GRU}\left(x_{t},\overset{\leftarrow }{h_{t+1}}\right)$$

### 3.2. Concatenation of Hidden States

The outputs from the forward and backward GRU are concatenated to form the final hidden state at time *t*:

$$h_{t}=\left[\vec{h_{t}};\overset{\leftarrow }{h_{t}}\right]$$

This dual representation allows the model to learn both historical and future dependencies, enhancing its ability to classify fault types and estimate locations accurately.

Multiple Bi-GRU layers are stacked to enable hierarchical feature extraction, with each layer learning progressively more abstract representations of the input data. This architecture ensures that both low-level patterns (e.g., transient spikes) and high-level temporal dynamics (e.g., fault propagation trends) are captured effectively.

The output layer maps the hidden states from the final Bi-GRU layer to fault classification and location probabilities. Before feeding into the fully connected layer, the three-dimensional hidden state tensor is reshaped to

[batchsize,sequencelength,2×hiddensize]

The reshaped tensor is then passed through a fully connected layer:

o=W·h+b

where

- *W* and *b* are learnable weights and biases.
- *o* represents the logits for fault classification.

### 3.3. Softmax Layer

A softmax activation function is applied to convert logits into class probabilities:

$${\hat{y}}_{i}=\frac{\exp(o_{i})}{\sum_{j=1}^{C}\exp\left(o_{j}\right)}$$

Here, *C* is the number of fault classes. The output probabilities correspond to the likelihood of each fault type, enabling the model to classify faults such as PTP, PPTG, and NPTG with high confidence.

### 3.4. Model Training and Optimization

The model is trained using the cross-entropy loss function, which measures the divergence between predicted probabilities and true labels:

$$L=-\frac{1}{N}\sum_{i=1}^{N}\sum_{j=1}^{C}y_{ij}\log{\hat{y}}_{ij}$$

where

- *N* is the number of samples in a batch.
- $y_{ij}$ is the ground truth label for sample *i* and class *j*.
- ${\hat{y}}_{ij}$ is the predicted probability for the same.

The Adam optimizer is employed to update model parameters, providing fast convergence with adaptive learning rates. A learning rate scheduler is used to reduce the learning rate when the validation loss plateaus, preventing overfitting.

## 4. Experimental Results

In an experiment, we compared Bi-GRU, Bi-LSTM, and transfer learning-based CNN models to evaluate their fault diagnosis performance in MVDC systems. These models were analyzed based on three key metrics: classification accuracy, diagnosis time, and robustness in noisy environments. The performance of each model was examined with a focus on their ability to detect and classify fault types and locations under various fault scenarios.

### 4.1. Data Preprocessing

To evaluate the fault diagnosis model, we simulated various fault scenarios in an MVDC system, assuming each fault initiates at 1.0 s. Voltage signals ($V_{P},V_{N}$) were extracted from 1.0 s to 1.5 s to capture both the transient response immediately after the fault onset and the post-fault period. A sliding window technique was then used to preprocess these voltage signals. The sliding window approach segments the continuous time-series data into overlapping windows, enabling enhanced temporal feature extraction and dataset augmentation. The parameters used for the sliding window technique include a window size of 2500 samples and a stride of 200 samples.

Regardless of the fault location, the diagnostic model analyzes voltage data measured at five fixed reference points along the MVDC transmission line: 10 km, 20 km, 30 km, 40 km, and 50 km. This consistent reference model ensures the uniform detection of waveform variations in voltage signals caused by faults. For instance, if a fault occurs near 3 km, the model references voltage data recorded at 10 km (and the other fixed points) to identify fault characteristics and estimate its location. Similarly, for faults occurring at 6 km, 10 km, or beyond, the same reference points are used. This consistent reference-point strategy enables the uniform detection of fault-induced voltage variations, enhancing the model’s generalization capability and ensuring robust and reliable fault detection and localization.

The validation process tests the model’s performance at locations not included in the training data, specifically at 6 km, 16 km, 26 km, 36 km, and 46 km. In contrast, the training dataset comprises fault scenarios from other locations, such as 3 km, 10 km, 13 km, 20 km, and 23 km, to develop a comprehensive fault diagnosis model. This data configuration focuses on evaluating the model’s ability to accurately detect and localize faults even at unseen locations.

The resulting dataset sizes after applying the sliding window technique are summarized in Table 2.

#### 4.1.1. Bi-GRU

The Bi-GRU model used for fault diagnosis is structured to process time-series data efficiently while capturing temporal dependencies in both forward and backward directions. The model’s configuration includes the following components.

The input layer processes data with five features, representing voltage signal measurements collected from fixed reference points along the MVDC transmission line (e.g., 10 km, 20 km, etc.). These features are essential for detecting location-based variations in voltage signals caused by faults.

**Figure 6:**
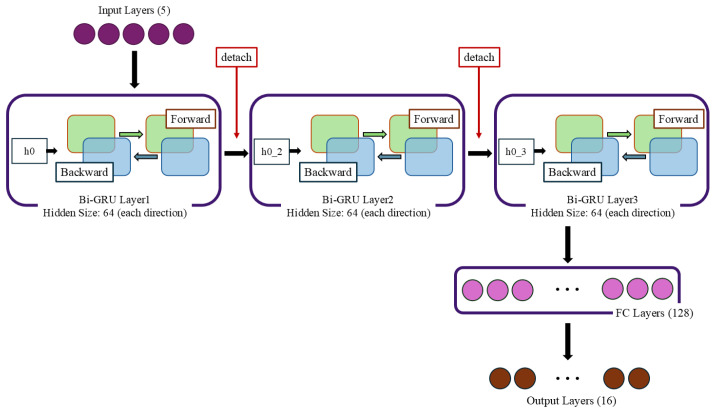
The structure of the Bi-GRU model, showing bidirectional layers and feature extraction.

The core of the model consists of two Bi-GRU layers, each with 64 hidden units in both forward and backward directions. This configuration enables the model to extract relevant temporal features while retaining critical bidirectional dependencies. The hidden state generated by the first Bi-GRU layer is passed to the third layer, ensuring a hierarchical extraction of patterns. The architectural structure of the Bi-GRU, including its bidirectional layers and hierarchical feature extraction capability, is illustrated in Figure 6.

Following the Bi-GRU layers, the output is fed into a fully connected (FC) layer with 128 neurons. This layer serves as a bridge between the extracted temporal features and the final classification. The last layer of the model, the output layer, generates 16 output classes corresponding to fault types and locations. A softmax activation function is applied to the output, converting logits into probabilities for each fault class.

To train the model effectively, the hyperparameters listed in Table 3 were used. These hyperparameters were selected to ensure efficient learning and optimal performance in detecting fault types and locations.

As shown in Table 3, the Adam optimizer was employed to ensure adaptive learning rates during training, while the cross-entropy loss function was utilized to handle the multi-class classification problem. The batch size of 64 and 50 training epochs were chosen to balance computational efficiency with model performance.

This structured approach ensures robust learning, allowing the Bi-GRU model to generalize effectively across diverse fault scenarios. By leveraging its capability to model temporal dependencies in a bidirectional manner, the model achieves high accuracy in detecting fault types and locations while maintaining computational efficiency.

#### 4.1.2. Bi-LSTM

The Bi-LSTM model was designed to capture bidirectional temporal dependencies in fault signals, similar to Bi-GRU [38]. It consists of three stacked Bi-LSTM layers, each containing 64 hidden units. The architecture used normalized 3D tensors as input, representing sequences of voltage signals ($V_{P},V_{N}$) sampled from the DC transmission line. The final layer of the Bi-LSTM was a fully connected layer with 16 output classes and a softmax activation function, mapping the learned features to fault types and locations. Despite its strong temporal modeling capabilities, the additional gates in LSTM introduced higher computational complexity, resulting in slower diagnosis times.

#### 4.1.3. Transfer Learning with CNN

In this study, a CNN model based on MobileNetV2 with transfer learning was designed to effectively analyze fault signals [39,40]. First, wavelet transforms were applied to signals measured at 10 km, 20 km, 30 km, 40 km, and 50 km, generating three-channel images of size 224 × 224 × 3 that match the input requirements of MobileNetV2 for transfer learning. Specifically, the data from 10 km and 20 km were assigned to the R channel, those from 30 km and 40 km were mapped to the G channel, and those from 50 km were placed in the B channel. Next, the pre-trained convolutional layers were fine-tuned to effectively capture the characteristics of fault signals. Finally, the original fully connected layer was removed and replaced with a new layer consisting of 512 neurons. A Softmax activation function was then applied to estimate the probability of each class, thereby addressing the multi-class classification problem.

**Figure 7:**
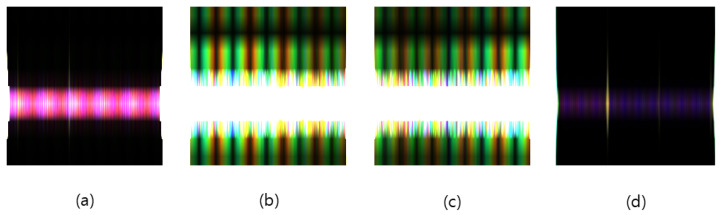
Continuous Wavelet Transform (CWT) for CNN fault classification performance for (**a**) Normal, (**b**) NPTG, (**c**) PPTG, and (**d**) PTP fault types.

Figure 7 shows Continuous Wavelet Transform (CWT) representations for various MVDC fault scenarios (Normal, NPTG, PPTG, and PTP) used in the CNN model. By converting voltage signals into time–frequency images, the CNN effectively identifies unique spectral patterns associated with each fault type.

**Figure 8:**
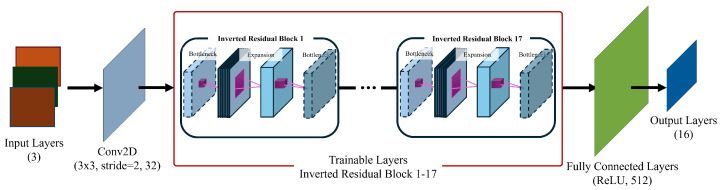
The architecture of the adapted MobileNetV2 CNN for MVDC fault diagnosis.

To further clarify the architectural modifications of the CNN model, Figure 8 presents the structural diagram of the adapted MobileNetV2 configuration. This diagram highlights the key adjustments made to the input layer and the fully connected layer, ensuring compatibility with the fault diagnosis model.

### 4.2. Comparison Analysis

The models were evaluated based on four key metrics: classification accuracy, diagnosis time, robustness in noisy environments, fault detection rate (FDR), and false alarm rate (FAR). Classification accuracy measures the percentage of faults correctly identified by the model. Diagnosis time assesses the average time required to process and classify fault signals, reflecting the model’s efficiency. Robustness evaluates the ability of the model to maintain performance under noisy conditions, which is critical for its applicability in real-world scenarios. The FDR represents the proportion of actual fault samples correctly classified as faults, where a high FDR indicates that the model rarely misses actual faults. The FAR denotes the proportion of normal samples misclassified as faults, with a low FAR indicating that the model rarely generates false alarms for normal data.

Table 4 presents a summary of the experimental results, comparing the Bi-GRU model against Bi-LSTM and Transfer-CNN.

The Bi-GRU model demonstrated superior performance, achieving the highest classification accuracy of 95.54% and the shortest diagnosis time of 1.3 ms. This highlights its capability to efficiently leverage bidirectional temporal modeling for fault detection and localization. The Bi-LSTM model achieved a classification accuracy of 89.8%, but its diagnosis time was longer at 2.1 ms due to the computational complexity introduced through additional gate mechanisms such as input, output, and forget gates. While these gates enhance the learning of temporal dependencies, they require more computations compared to Bi-GRU, making Bi-LSTM slightly less efficient for real-time applications.

In contrast, the CNN model showed limited performance in analyzing sequential signals due to its inability to directly model temporal dependencies. It achieved the lowest classification accuracy of 87.49% and the longest diagnosis time of 5.34 ms. Additionally, the CNN required a preprocessing step to convert fault signals into image-like formats, which posed challenges for real-time fault diagnosis and further increased the diagnosis time.

In addition to classification accuracy, the FDR and FAR were used as essential metrics for evaluating model performance. The Bi-GRU model achieved an FDR of 95.13% and an FAR of 0%, demonstrating excellent fault detection capabilities without generating false alarms. The Bi-LSTM model also showed strong performance, with an FDR of 89.2% and an FAR of 0%. However, the CNN model exhibited a relatively lower FDR of 86.8% while maintaining an FAR of 0%. This indicates that while the CNN is less effective in fault detection compared to the other models, it still avoids false alarms entirely.

From the perspective of diagnosis time, Bi-LSTM’s additional gate mechanisms contribute to precise temporal dependency learning but result in a longer average diagnosis time of 2.1 ms compared to Bi-GRU’s 1.3 ms. The CNN model recorded the longest diagnosis time of 5.34 ms due to its preprocessing requirements and inability to directly handle temporal dependencies. These factors make the CNN model less suitable for real-time fault diagnosis applications compared to Bi-GRU and Bi-LSTM.

**Figure 9:**
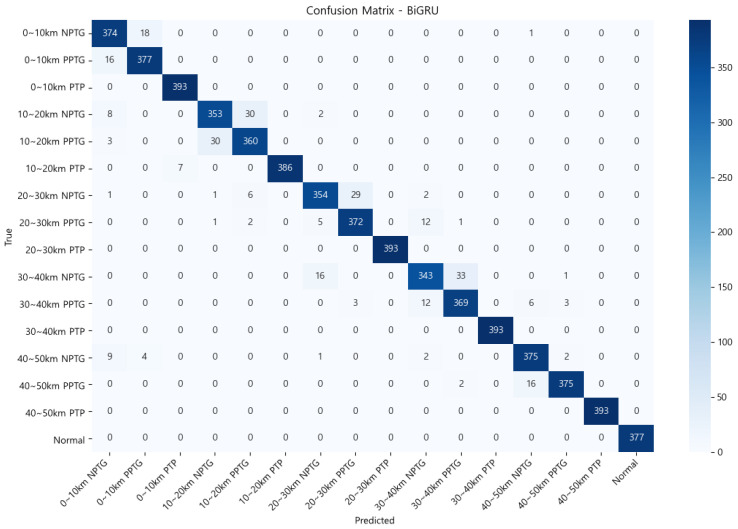
Confusion matrix of the Bi-GRU model for Normal, PTP, PPTG, and NPTG fault types.

Figure 9 illustrates the confusion matrix of the Bi-GRU model, showing its classification performance for various fault scenarios, including Normal, PTP, PPTG, and NPTG fault types. The matrix highlights the model’s ability to differentiate between fault types effectively and emphasizes its robustness under diverse conditions.

In conclusion, the Bi-GRU model effectively addresses the limitations of conventional models, providing superior performance in accuracy and diagnosis time. Its robustness under noisy conditions validates its practicality for real-world applications in MVDC systems.

## 5. Conclusions

This study presented a Bi-GRU-based fault diagnosis model specifically designed for MVDC systems. By addressing fault type and location identification as a multi-classification problem, the proposed method demonstrated significant advancements in diagnostic precision, efficiency, and robustness. The Bi-GRU model effectively captured bidirectional temporal dependencies, enabling the accurate identification of fault types, including PTP, PPTG, and NPTG, as well as the precise localization of fault points along the MVDC transmission line.

This research highlighted several notable achievements. First, the model achieved a diagnostic accuracy of 95.54% and an average fault diagnosis time of 5.9 ms, outperforming traditional methods such as Bi-LSTM and CNN models. This underscores the model’s capability to handle the unique temporal dynamics of fault signals. Additionally, the model exhibited robustness against noise, validating its reliability under real-world operational conditions. Furthermore, the Bi-GRU model showcased robust performance under various fault scenarios, underscoring its versatility and adaptability in managing diverse operational conditions.

The findings of this study underline the potential of Bi-GRU-based methods to overcome the challenges associated with MVDC systems, particularly those involving extended transmission distances and complex fault patterns. Moreover, the scalability of the proposed model was validated through its performance across diverse operating conditions and system configurations, indicating its applicability to various MVDC scenarios.

However, there remains scope for future research to expand upon these findings. Enhancing the dataset to include a wider variety of fault scenarios and MVDC architectures could improve the model’s generalizability. Additionally, exploring real-time hardware implementations will be crucial to assess the feasibility of deploying this model in practical settings. Furthermore, through the exploration of a variety of strategies to maximize the potential of Bi-GRU, it becomes possible to further refine temporal feature analysis and enhance fault diagnosis performance.

Future work will extend the application of this model to a four-terminal MVDC system, further validating its robustness and scalability under more complex network topologies. This extension will focus on addressing additional challenges such as increased fault detection complexity and network dynamics in multi-terminal configurations.

In addition, efforts are underway to construct a test bed to evaluate the proposed method in a real-world environment. Before applying the method to the test bed, Hardware-in-the-Loop Simulation (HILS) will be conducted to ensure its reliability and performance under practical conditions.

In conclusion, this research advances intelligent fault diagnosis methodologies for MVDC systems, contributing to improved grid reliability and supporting the integration of renewable energy solutions. By addressing critical challenges in fault detection and diagnosis, the proposed model lays a solid foundation for the development of future intelligent protection systems, paving the way for a more sustainable and efficient energy landscape.

## Figures and Tables

**Table 1:** MVDC parameters for training and testing.

Devices	Description	Parameters	Value
Voltage Source Converter	Capacity [MW]	$P_{\mathrm{rated}}$	20
	DC rated voltage [kV]	$V_{\mathrm{dc},\;\mathrm{rated}}$	±35
	AC rated voltage [kV]	$V_{\mathrm{ac},\;\mathrm{rated}}$	40
	Switching frequency [Hz]	$f_{\mathrm{sw}}$	1640
	DC capacitance [mH]	$C_{\mathrm{dc},\;\mathrm{cap}}$	3.4
DC line	Resistance [Ω/km]	$R_{\mathrm{dc}}$	0.015
	Inductance [mH/km]	$L_{\mathrm{dc}}$	0.3
	Capacitance [μF/km]	$C_{\mathrm{dc}}$	13.75
	Length [km]	*L*	50
	Fault Resistance [Ω]	$R_{\mathrm{fault}}$	0.001

**Table 2:** Sliding window configuration and dataset statistics.

Parameter	Value
Window Size (samples)	2500
Stride (samples)	200
**Dataset**	**Number of Samples**
Training Set	12,183
Validation Set	6288

**Table 3:** Training configuration for Bi-GRU model.

Parameter	Value
Optimizer	Adam
Loss Function	Cross-Entropy Loss
Learning Rate	0.001
Batch Size	64
Epochs	50

**Table 4:** Fault diagnosis performance.

Model	Diagnosis Time (ms)	Accuracy (%)	FDR (%)	FAR (%)
CNN (MobileNetV2)	5.34	87.49	86.8	0
Bi-LSTM	2.1	89.8	89.2	0
Bi-GRU	1.3	95.54	95.13	0

## Data Availability

The original contributions presented in the study are included in the article; further inquiries can be directed to the corresponding author.

## References

[1] Alam M.S., Al-Ismail F.S., Salem A., Abido M.A. (2020). High-level penetration of renewable energy sources into grid utility: Challenges and solutions. IEEE Access.

[2] Attanayake K., Wickramage I., Samarasinghe U., Ranmini Y., Ehalapitiya S., Jayathilaka R., Yapa S. (2024). Renewable energy as a solution to climate change: Insights from a comprehensive study across nations. PLoS ONE.

[3] Warner K.J., Jones G.A. (2017). The climate-independent need for renewable energy in the 21st century. Energies.

[4] Sinsel S.R., Riemke R.L., Hoffmann V.H. (2020). Challenges and solution technologies for the integration of variable renewable energy sources—A review. Renew. Energy.

[5] Mathew E.C., Das A. (2020). Integration of renewable energy sources with MVDC network. Proceedings of the 2020 IEEE International Conference on Power Electronics, Drives and Energy Systems (PEDES).

[6] Shafiullah M., Ahmed S.D., Al-Sulaiman F.A. (2022). Grid integration challenges and solution strategies for solar PV systems: A review. IEEE Access.

[7] Coffey S., Timmers V., Li R., Wu G., Egea-Àlvarez A. (2021). Review of MVDC applications, technologies, and future prospects. Energies.

[8] Yuan C., Haj-Ahmed M.A., Illindala M.S. (2015). Protection strategies for medium-voltage direct-current microgrid at a remote area mine site. IEEE Trans. Ind. Appl..

[9] Jin Z., Meng L., Guerrero J.M., Han R. (2017). Hierarchical control design for a shipboard power system with DC distribution and energy storage aboard future more-electric ships. IEEE Trans. Ind. Inform..

[10] Doerry N., Amy J., Krolick C. (2015). History and the status of electric ship propulsion, integrated power systems, and future trends in the US Navy. Proc. IEEE.

[11] Bosich D., Vicenzutti A., Pelaschiar R., Menis R., Sulligoi G. Toward the future: The MVDC large ship research program. Proceedings of the 2015 AEIT International Annual Conference (AEIT).

[12] Kim J.M., Han B.G., Lee H.D., Cho S.D., Rho D.S. (2023). A Study on Protection Coordination Operation Method of ±35 kV Hybrid MVDC Distribution System. J. Korea Acad.-Ind. Coop. Soc..

[13] Ma Z., Li R., Lürkens P., Han M., Kim S.N. (2020). Medium Voltage Direct Current (MVDC) Grid Feasibility Study.

[14] Chaudhuri N., Chaudhuri B., Majumder R., Yazdani A. (2014). Multi-Terminal Direct-Current Grids: Modeling, Analysis, and Control.

[15] Jiang S., Fan C., Huang N., Zhu Y., He M. (2018). A fault location method for DC lines connected with DAB terminal in power electronic transformer. IEEE Trans. Power Deliv..

[16] Xiao Z., Zheng X., He Y., Tai N., Fan C., Huang N., Jiang S. (2021). An Accurate Analysis Method for Transient Characteristics of DC Line Faults in Voltage Source Converter-Based DC Systems. IET Gener. Transm. Distrib..

[17] Monadi M., Gavriluta C., Luna A., Candela J.I., Rodriguez P. (2016). Centralized protection strategy for medium voltage DC microgrids. IEEE Trans. Power Deliv..

[18] Emhemed A.A., Fong K., Fletcher S., Burt G.M. (2016). Validation of fast and selective protection scheme for an LVDC distribution network. IEEE Trans. Power Deliv..

[19] Dai Z., Ge H., Yan S., Jiao Y., Chen X. (2017). Effects of grounding mode on fault characteristics in flexible DC distribution system. Power Syst. Technol..

[20] Feng W., Yuan C., Shi Q., Dai R., Liu G., Wang Z., Li F. (2020). Graph computing based distributed parallel power flow for AC/DC systems with improved initial estimate. J. Mod. Power Syst. Clean Energy.

[21] Dai X., Gao Z. (2013). From model, signal to knowledge: A data-driven perspective of fault detection and diagnosis. IEEE Trans. Ind. Inform..

[22] Shoaib M.A., Khan A.Q., Mustafa G., Gul S.T., Khan O., Khan A.S. (2021). A framework for observer-based robust fault detection in nonlinear systems with application to synchronous generators in power systems. IEEE Trans. Power Syst..

[23] Song R., Sepehri N. Fault detection and isolation in fluid power systems using a parametric estimation method. Proceedings of the IEEE CCECE2002. Canadian Conference on Electrical and Computer Engineering, Cat. No. 02CH37373.

[24] Wang Z., Xu A., Yang Z. Application of Model-based and Data-driven Techniques in Fault Diagnosis. Proceedings of the 2007 8th International Conference on Electronic Measurement and Instruments.

[25] Silva K.M., Souza B.A., Brito N.S. (2006). Fault detection and classification in transmission lines based on wavelet transform and ANN. IEEE Trans. Power Deliv..

[26] Rai P., Londhe N.D., Raj R. (2021). Fault classification in power system distribution network integrated with distributed generators using CNN. Electr. Power Syst. Res..

[27] Tayeb E.B.M. (2013). Faults detection in power systems using artificial neural network. Am. J. Eng. Res..

[28] Shadi M.R., Ameli M.-T., Azad S. (2022). A real-time hierarchical framework for fault detection, classification, and location in power systems using PMUs data and deep learning. Int. J. Electr. Power Energy Syst..

[29] Pérez-Molina M.J., Larruskain D.M., Eguía López P., Buigues G. (2020). Challenges for protection of future HVDC grids. Front. Energy Res..

[30] Wang Y., Zheng D., Jia R. (2022). Fault diagnosis method for MMC-HVDC based on Bi-GRU neural network. Energies.

[31] Anttila S., Döhler J.S., Oliveira J.G., Boström C. (2022). Grid forming inverters: A review of the state of the art of key elements for microgrid operation. Energies.

[32] Askarov A., Rudnik V., Ruban N., Radko P., Ilyushin P., Suvorov A. (2024). Enhanced Virtual Synchronous Generator with Angular Frequency Deviation Feedforward and Energy Recovery Control for Energy Storage System. Mathematics.

[33] Lee H.-D., Kim K.Y., Kim M.S., Rho D.S. (2021). A study on economic evaluation modeling of MVDC distribution system for hosting capacity of PV system. J. Korea Acad.-Ind. Coop. Soc..

[34] Muniappan M. (2021). A Comprehensive Review of DC Fault Protection Methods in HVDC Transmission Systems. Prot. Control Mod. Power Syst..

[35] Nanayakkara O.M.K., Rajapakse A.D., Wachal R. (2011). Location of DC line faults in conventional HVDC systems with segments of cables and overhead lines using terminal measurements. IEEE Trans. Power Deliv..

[36] Cho K., Van Merriënboer B., Gulcehre C., Bahdanau D., Bougares F., Schwenk H., Bengio Y. (2014). Learning phrase representations using RNN encoder-decoder for statistical machine translation. arXiv.

[37] Schuster M., Paliwal K.K. (1997). Bidirectional recurrent neural networks. IEEE Trans. Signal Process..

[38] Hochreiter S. (1997). Long Short-term Memory. Neural Comput..

[39] Krizhevsky A., Sutskever I., Hinton G.E. (2012). Imagenet classification with deep convolutional neural networks. Advances in Neural Information Processing Systems, Proceedings of the 25th Conference on Neural Information Processing Systems (NeurIPS 2012), Lake Tahoe, NV, USA, 3–8 December 2012.

[40] Sandler M., Howard A., Zhu M., Zhmoginov A., Chen L.C. Mobilenetv2: Inverted residuals and linear bottlenecks. Proceedings of the IEEE Conference on Computer Vision and Pattern Recognition (CVPR).

